# 

**Published:** 1889-08

**Authors:** 


					﻿Vol IX.
AUGUST, 1889..
'No.-8.
IPHE
HOMEOPATHIC pH^lClAJI,
A Monthly Journal of Homoeopathic Materia Medica
and Clinical Medicine.
“ He is a freeman whom truth makes free, and all are slaves beside."
u Seek the Truth: come whence it may, cost what it will.”
EDITED AND PUBLISHED BY
Edmund j.	M.
AND
: WALTER ML. JAMES, ME. D.
PHILADELPHIA :
No. 1123 ST’RTTCK STREET.
london
ALFRED HEATH & CO., Sole Agents foe Great Britain,
114 Ebury Street, Eaton Square.
early Subscription, $2.50. invariably in advance. Single numbers, 25 cts.
Single numbers containing the Repertory Supplement, 50 cts.
Entered at the Philadelphia Poet-Office as Second-Class Mail Matter.
				

